# RACE, DEATH OF A SIBLING, AND DEMENTIA RISK AT LATE ADULTHOOD

**DOI:** 10.1093/geroni/igz038.3011

**Published:** 2019-11-08

**Authors:** Minle Xu

**Affiliations:** The University of Texas at Austin, Austin, United States

## Abstract

The death of a family member is a stressful life event that compromises health and well-being. Considerable research has documented the detrimental health effects of parental, child, and spousal death. However, much less is known with regard to the health consequences of sibling death especially in late adulthood. Relationship with sibling is one of longest and intimate social relationships and the death of a sibling can be a devastating life event especially for older adults as they are more vulnerable to adverse effect of stress. As sibling death is more prevalent in late adulthood, it is important to examine whether sibling death increases risks of dementia which has become a public health concern due to its deleterious effects on individuals, families, and societies. Therefore, this study investigates the association between sibling death and dementia incidence in later life by using longitudinal data from the Health and Retirement Study. Results from discrete-time hazard models show that respondents who experienced the death of a sibling between 1992 and 2000 are more likely to develop dementia during follow-up. This positive association between the death of a sibling and dementia incidence remains unchanged after accounting for respondents’ health status before sibling death and shared family social status during childhood. Further analyses indicate that psychological distress, health behaviors, and health status cannot explain the relationship between sibling death and dementia incidence. In addition, the association of sibling death with dementia incidence is similar for non-Hispanic whites and non-Hispanic blacks.

